# Phase I study of Carzelesin (U-80,244) given (4-weekly) by intravenous bolus schedule

**DOI:** 10.1038/sj.bjc.6690232

**Published:** 1999-03

**Authors:** A Awada, C J A Punt, M J Piccart, O Van Tellingen, L Van Manen, J Kerger, Y Groot, J Wanders, J Verweij, D J Th Wagener, on behalf of the EORTC Early Clinical Studies Group and EORTC NDDO

**Affiliations:** Institut Jules Bordet, Institut Jules Bordet, Bd de Waterloo 125, Brussels, 1000, Belgium; University Hospital, Geert Grooteplein 8, Box 9101, Nijmegen, 6500 HB Nijmegen, The Netherlands; The Netherlands Cancer Institute (Antoni van Leeuwenhoek Huis), The Netherlands Cancer Institute, Plesmanlaan 121, Amsterdam, 1066 CX Amsterdam, The Netherlands; European Organization for Research and Treatment of Cancer – New Drug Development Office, European Organization for Research and Treatment of Cancer, PO Box 7057, Amsterdam, The Netherlands; Rotterdam Cancer Institute, Groene Hilledijk 301, Rotterdam, 3075 EA Rotterdam, The Netherlands

**Keywords:** Carzelesin, phase I, alkylating agent, pharmacokinetics, phamacodynamics

## Abstract

Carzelesin is a cyclopropylpyrroloindole analogue which acts as a DNA-sequence-specific alkylating agent. In this phase I study, Carzelesin was given as a 4-weekly 10 min IV infusion to 51 patients with advanced solid tumours. Patients received a median of two courses (range 1–5) at one of nine dose levels: 24, 48, 96, 130, 150, 170, 210, 250 and 300 μg m^−2^. According to NCI-CTC criteria, non-haematological toxicities (grade 1/2) included fever, nausea and vomiting, mucositis and anorexia, none of which was clearly dose related. The dose-limiting toxicity was haematological and consisted mainly of neutropenia and to a lesser extent thrombocytopenia. From the dose level 150 μg m^−2^, the haematological toxicity (particularly thrombocytopenia) was delayed in onset, prolonged and cumulative in some patients. In several courses, double WBC nadirs occurred. The maximum tolerated dose for a single course was 300 μg m^−2^. From the dose level 170 μg m^−2^, the intended dose intensity could not be delivered to most patients receiving > 2 courses owing to cumulative haematological toxicity. The dose level with the best dose intensity for multiple courses was 150 μg m^−2^. The pharmacokinetics of Carzelesin and its metabolites (U-76,073; U-76,074) have been established in 31 patients during the first course of treatment using a HPLC method. Carzelesin exhibited linear pharmacokinetics. The concentration of U-76,074 (active metabolite) extended above the lower limit of quantitation (1 ng ml^−1^) for short periods of time and only at the higher dose levels. There was no relationship between neutropenia and the AUC of the prodrug Carzelesin, but the presence of detectable plasma levels of the active metabolite U-76,074 was usually associated with a substantial decrease in ANC values. © 1999 Cancer Research Campaign


*British Journal of Cancer* (1999) **79**(9/10), 1454–1461
© 1999 Cancer Research Campaign
Article no. bjoc.1998.0232

### Phase I study of Carzelesin (U-80,244) given (4-weekly) by intravenous bolus schedule

**A Awada[1], CJA Punt[2], MJ Piccart[1], O Van Tellingen[3], L Van Manen[2], J Kerger[1], Y Groot[4], J Wanders[4], J Verweij[5] and DJTh Wagener[2]**

[1]Institut Jules Bordet, Bd de Waterloo 125, 1000 Brussels, Belgium; [2]University Hospital, Geert Grooteplein 8, Box 9101, 6500 HB Nijmegen, The Netherlands; [3]The Netherlands Cancer Institute (Antoni van Leeuwenhoek Huis), Plesmanlaan 121, 1066 CX Amsterdam, The Netherlands; [4]European Organization for Research and Treatment of Cancer – New Drug Development Office, PO Box 7057, Amsterdam, The Netherlands; [5]Rotterdam Cancer Institute, Groene Hilledijk 301, 3075 EA Rotterdam, The Netherlands; on behalf of the EORTC Early Clinical Studies Group and EORTC NDDO

**Summary** Carzelesin is a cyclopropylpyrroloindole analogue which acts as a DNA-sequence-specific alkylating agent. In this phase I study, Carzelesin was given as a 4-weekly 10 min IV infusion to 51 patients with advanced solid tumours. Patients received a median of two courses (range 1–5) at one of nine dose levels: 24, 48, 96, 130, 150, 170, 210, 250 and 300 μg $m^{-2}$. According to NCI-CTC criteria, non-haematological toxicities (grade 1/2) included fever, nausea and vomiting, mucositis and anorexia, none of which was clearly dose related. The dose-limiting toxicity was haematological and consisted mainly of neutropenia and to a lesser extent thrombocytopenia. From the dose level 150 μg $m^{-2}$, the haematological toxicity (particularly thrombocytopenia) was delayed in onset, prolonged and cumulative in some patients. In several courses, double WBC nadirs occurred. The maximum tolerated dose for a single course was 300 μg $m^{-2}$. From the dose level 170 μg $m^{-2}$, the intended dose intensity could not be delivered to most patients receiving > 2 courses owing to cumulative haematological toxicity. The dose level with the best dose intensity for multiple courses was 150 μg $m^{-2}$. The pharmacokinetics of Carzelesin and its metabolites (U-76,073; U-76,074) have been established in 31 patients during the first course of treatment using a HPLC method. Carzelesin exhibited linear pharmacokinetics. The concentration of U-76,074 (active metabolite) extended above the lower limit of quantitation (1 ng $ml^{-1}$) for short periods of time and only at the higher dose levels. There was no relationship between neutropenia and the AUC of the prodrug Carzelesin, but the presence of detectable plasma levels of the active metabolite U-76,074 was usually associated with a substantial decrease in ANC values.

**Keywords:** Carzelesin; phase I; alkylating agent; pharmacokinetics, phamacodynamics

Carzelesin (U-80,244, Figure 1A) is a cyclopropylpyrroloindole (CPI) prodrug analogue of CC-1065. CC-1065 is a natural product isolated from the soil organism *Streptomyces zelensis*, which proved to be an extremely potent cytotoxic compound. However, its clinical development as an antitumour agent was halted because preclinical toxicology studies revealed that this agent caused delayed fatal toxicity in mice and rabbits at sub-therapeutic doses (McGovren et al, 1984). CPI drugs are a class of compounds with unique DNA interactive properties. These agents fit into the minor groove region of DNA, and the CPI function in the molecule mediates a covalent binding to the $N^3$ position of adenine in A-T rich regions, thus forming DNA adducts in a sequence selective fashion (Hurley et al, 1984; Reynolds et al, 1985).

Three new CPIs have now been developed. Adozelesin is most closely related to CC-1065 as it already possesses the active CPI-moiety (Figure 1A). Bizelesin differs from the classical CPI drugs in that it contains two chloromethyl functions, which are both converted (via intramolecular rearrangements) to the CPI alkylating species that interact with DNA. Consequently, this agent is capable of forming DNA interstrand cross-links (Lee et al, 1991). Carzelesin was designed to be an inactive prodrug as earlier studies with CC-1065 had revealed that DNA alkylation by CPI drugs occurred in a rapid fashion (Reynolds et al, 1985; Warpehoski et al, 1988). Therefore, efforts were directed to modulate the alkylation rate by the preparation of CPI prodrugs that would require chemical or enzymatic conversion. The activation of Carzelesin is a two-step reaction with hydrolysis of the phenylurethane substituent to form the intermediate compound U-76,073, followed by ring closure to form the active product U-76,074 (Figure 1B). Although Carzelesin was found to be less potent than adozelesin against tumour cells cultured in vitro, it proved to be more efficacious in a broad panel of murine and human tumour xenografts (Li et al, 1992). This higher efficacy may be related to the pharmacologic features of the compound. Owing to the high reactivity of the CPI moiety, the inactive prodrug may be able to penetrate (target) tissues more effectively than an active CPI drug, before conversion to the active compound.

In preclinical studies, Carzelesin was highly effective against Lewis lung carcinoma, B16 melanoma, colon 38 carcinoma, colon CX1 adenocarcinoma, lung LX-1 tumour, ovarian 2780 and prostatic DU-145 carcinoma (Li et al, 1992; Houghton et al, 1995). Carzelesin toxicity in preclinical studies occurred in bone marrow, kidney, liver and spleen (EORTC-NDDO, 1992).

*Received 20 April 1998*
*Revised 31 August 1998*
*Accepted 13 October 1998*

*Correspondence to:* A Awada, Jules Bordet Institute, Chemotherapy Unit, Rue Héger-Bordet 1, 1000 Brussels, Belgium

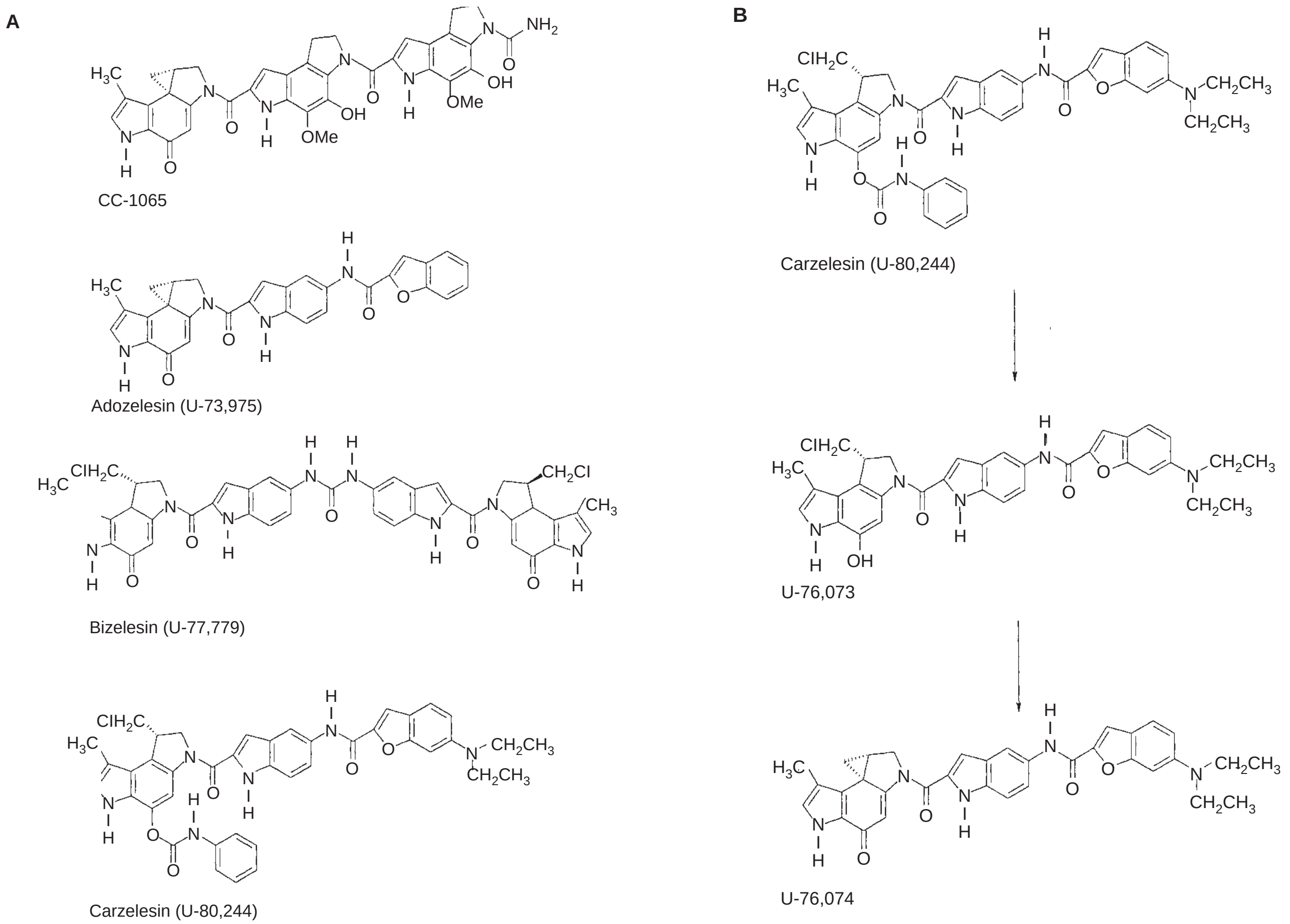

**Figure 1** (**A**) Molecular structures of CC-1065, adozelesin (U73,975), bizelesin (U77,779), carzelesin (U-80,244). (**B**) Molecular structures of carzelesin (U-80,244), the intermediate metabolite U-76,073 and the active metabolite U-76,074

As all three compounds displayed promising results in preclinical testing, they were selected for phase I testing. Two phase I clinical trials with adozelesin have been completed in the USA (Shamdas et al, 1994; Fleming et al, 1994). The phase I trial with bizelesin is still in the planning stage by the NCI of the USA. Carzelesin has been selected for clinical development in Europe under the framework of the EORTC.

Co-ordinated by the EORTC-NDDO, two multicentre phase I studies with Carzelesin were started in Europe. The patients received the drug by a brief (10 min) IV infusion given once every 4 weeks or in a daily × 5 schedule repeated every 4 weeks (Wolf et al, 1996).

In this report we describe the phase I clinical study with Carzelesin administered as a single dose intravenously every 4 weeks. The aims of this study were to determine the toxic effects of Carzelesin, the maximum tolerated dose (MTD), the pharmacokinetics and to document any antitumour activity.

### PATIENTS AND METHODS

### Patient population

Patients with a histological diagnosis of solid tumour no longer amenable to established forms of treatments were eligible. Other inclusion criteria included: age 18–75 years, ECOG performance status ≤ 2, white blood cell count ≥ 4 × $10^9$ $l^{-1}$, platelet count ≥ 100 × $10^9$ $l^{-1}$, bilirubin < 2 mg $dl^{-1}$, serum creatinine ≤ 1.4 mg $dl^{-1}$, no clinical signs of brain involvement or leptomeningeal disease, no prior chemotherapy or extended field radiotherapy for at least 4 weeks prior to study entry. All patients gave written informed consent and the protocol was approved by the Ethics Committee of the two participating institutions as well as the Protocol Review Committee of the EORTC.

### Screening and follow-up studies

Prior to study, each patient underwent a complete history and physical examination. Pretreatment laboratory investigations included a complete blood count with leukocyte differential, standard serum biochemistry and coagulation studies, urinalysis and creatinine clearance, chest X-ray and electrocardiogram. Appropriate radiological examinations for tumour measurements were obtained before study entry. During the study, haematology and biochemistry assessments were repeated weekly, or twice weekly in all patients at that and subsequent doses following significant haematological or biochemical toxicity (≥ grade 2) in any patient.

© Cancer Research Campaign 1999

### Treatment: starting dose, drug administration, drug dose escalation

Preclinical toxicology in the mouse showed that the $LD_{10}$ was 280 µg $kg^{-1}$, which is equivalent to a $MELD_{10}$ of 840 µg $m^{-2}$. It is generally accepted that 1/10 $MELD_{10}$ provides a safe starting dose for phase I clinical trials, as long as it is not toxic to a second species, i.e. the rat. As for Carzelesin, a difference in species sensitivity was seen, a dose level at 1/35 $MELD_{10}$ was investigated in toxicological studies and was considered as a safe starting dose for human single-dose clinical trials. Therefore, the starting dose of this phase I trial was 24 µg $m^{-2}$.

Carzelesin was supplied by Pharmacia & Upjohn Company (Kalamazoo, Michigan, USA) in 2-ml vials containing a drug concentration of 250 µg $ml^{-1}$ in a non-aqueous vehicle, comprised of a 2:1 mixture of polyethylene glycol 400 and ethanol, with 10% Tween 80 (PET) (Jonkman-de Vries et al, 1994). This pharmaceutical preparation was kept frozen at – 30°C and protected against light.

For use the Carzelesin concentrate was diluted 10-fold with a PET-vehicle to a concentration of 25 µg $ml^{-1}$. Next, the appropriate volume containing the prescribed dose was diluted with 5% dextrose in water (D5W) to a final volume of 20 ml. Carzelesin was administered using a PVC bag in one centre and a syringe pump in the other, according to routine local practice. The drug solution was infused intravenously at a dose rate of 2 ml $min^{-1}$ (10 min infusion time). Carzelesin was administered every 4 weeks, with delays for toxicity recovery.

Doses were escalated in decreasing rates according to the Fibonacci scheme and modified to reflect the emerging results of a parallel phase I study (daily × 5 q 28 d) (Wolf et al, 1996). A minimum of three patients receiving at least four evaluable courses were to be evaluated at non-toxic dose levels. If significant (≥ grade II) toxicity was observed at a given dose level, more patients were entered at that dose level. At each dose level, 1 week, or 2 weeks at higher dose levels, was necessary between the entry of the first and the next two patients. The dose limiting toxicity (DLT) was determined. The MTD was defined as the highest dose that can be safely administered to a patient population producing, manageable and reversible toxicity of CTC grade 3, or grade 4 in case of haematological toxicity in at least 2/6 patients. Discontinuation of treatment was required for non-compliance of the patient, excessive toxicity and progressive disease.

**Table 1** Patient characteristics (*n* = 51)

| | |
|---|---|
| Male/female | 34/17 |
| Median age (range) | 53 (21–71) |
| Median PS (range) | 1 (0–2) |
| Previous therapy | |
| Chemotherapy (CT) | 25 |
| Median no. of prior CT regimens (range) | 2 (1–4) |
| Prior exposure to nitrosourea/mitomycin | 7/1 |
| Radiotherapy (RT) | 3 |
| CT + RT | 21 |
| No treatment | 2 |
| Tumour type | |
| Colorectal | 13 |
| Melanoma | 8 |
| Head and neck | 7 |
| Sarcoma | 5 |
| Unknown origin | 4 |
| Renal | 3 |
| Other | 11 |

### Assessment of toxicity and response

Patients were evaluable for toxicity if they received at least one course of therapy. The evaluation period included all required observations until recovery from all toxicities associated with the first course of therapy. Toxicity was graded according to the common toxicity criteria of the National Cancer Institute (Bethesda, USA).

Patients were evaluable for antitumour activity if disease measurements were performed over at least an 8-week period from the first dose of therapy. Standard tumour measurement procedures were used including X-rays, CTscan, magnetic resonance and ultrasound imaging. Response was to be classified according to standard WHO criteria.

### Pharmacokinetics–pharmacodynamics study

A detailed report of the clinical pharmacokinetics of Carzelesin has been previously reported (Van Tellingen et al, 1998). In short, the pharmacokinetics have been established in 31 patients enrolled in this phase I study with at least two patients per dose level sampled during their first course of chemotherapy. Plasma levels of Carzelesin, and metabolites U-76,073 and U-76,074 (active metabolite) were determined by a selective method based on high-performance liquid chromatography (Van Tellingen et al, 1994a). Plasma concentration vs. time curves were fitted using the MW/Pharm software package. A one- or two-compartment infusion model was used to calculate the distribution and elimination half-lives of Carzelesin. The area under the concentration–time curve (AUC) of Carzelesin, U-76,073 and U-76,074 was calculated using the linear trapezoidal rule. The pharmacodynamics of the dose-limiting bone marrow toxicities were explored using plots of percentage decrease (%decr) of granulocytes (ANC), white blood cells (WBC) or platelets defined as

%decr = (pretreatment value – value of the nadir)/pretreatment value × 100% vs. the AUC of Carzelesin or its active metabolite (U-76,074)

### RESULTS

### Patient characteristics

Fifty-one eligible patients entered the trial from December 1992 to October 1995. The demographic and treatment characteristics of the study population are shown in Table 1.

### Carzelesin administration

A total of nine dose levels up to 300 µg $m^{-2}$ were investigated and 104 courses of Carzelesin were administered. The median number of courses given was 2 (1–5). Table 2 summarizes the treatment courses of Carzelesin.

### Haematological toxicity

Eighty-one of 104 courses were evaluable for haematological toxicity in 48 patients. Three patients were not evaluable owing to

 *© Cancer Research Campaign 1999*

**Table 2** Carzelesin: treatment courses

| **Dose level ($\mu$g m$^{-2}$)** | **No. of patients total/evaluable (*n* = 51)/(*n* = 48)** | **No. of courses total/evaluable (*n* = 104)/(*n* = 81)** | **No. of reduced courses (dose level)[a] (*n* = 17)** | **No. of courses with delayed recovery (no. of pts)[a] (*n* = 17)** |
|---|---|---|---|---|
| 24 | 4/4 | 6/6 | 0 | 0 |
| 48 | 3/3 | 9/7 | 0 | 0 |
| 96 | 5/5 | 8/8 | 0 | 0 |
| 130 | 6/5 | 10/9 | 0 | 0 |
| 150 | 6/6 | 17/14 | 3[b] (130) | 3 (1) |
| 170 | 11/9 | 19/16 | 0 | 4 (4) |
| 210 | 7/7 | 16/10 | 6[c] (96, 105, 150) | 5 (5) |
| 250 | 5/5 | 13/7 | 6[d] (130, 170) | 3 (3) |
| 300 | 4/4 | 6/4 | 2[e] (150) | 2 (2) |

[a]Due to haematological toxicity (courses delayed for more than 3 days). [b]1 patient; [c]4 patients; [d]3 patients; [e]2 patients.

Table 3 Carzelesin: Grade 3/4 haematological toxicity

| **Dose level ($\mu$g m$^{-2}$)** | **24** | **48** | **96** | **130** | **150** | **170** | **210** | **250** | **300** |
|---|---|---|---|---|---|---|---|---|---|
| No. of evaluable patients/courses | 4/6 | 3/7 | 5/8 | 5/9 | 6/14 | 9/16 | 7/10 | 5/7 | 4/4 |
| No. of toxic[a] patients[b] courses | 0 | 0 | 2/2 | 3/3 | 4/5 | 6/8 | 5/6 | 2/3 | 3/3 |
| Neutropenia grade 3/4 | | | | | | | | | |
| No.[b] of patients | 0 | 0 | 0/1 | 2/0 | 1/2 | 1/3 | 2/3 | 0/2 | 1/2 |
| No. of courses | 0 | 0 | 0/1 | 2/0 | 2/2 | 1/3 | 3/3 | 1/2 | 1/2 |
| Thrombocytopenia grade 3/4 | | | | | | | | | |
| No.[b] of patients | 0 | 0 | 1/0 | 2/1 | 1/1 | 3/2 | 0/2[c] | 1/1 | 1/1 |
| No. of courses | 0 | 0 | 1/0 | 2/1 | 1/1 | 5/2 | 0/2 | 1/1 | 1/1 |
| No. of toxic patients following the first course[a] | 0 | 0 | 2 | 0 | 2 | 4 | 4 | 2 | 3 |
| Neutropenia grade 3/4 | 0 | 0 | 0/1 | 0 | 0/1 | 0/2 | 3/1 | 1/1 | 1/2 |
| Thrombocytopenia grade 3/4 | 0 | 0 | 1/0 | 0 | 1/1 | 3/0 | 0/1[c] | 1/0 | 1/1 |

[a]Grade 3/4 neutropenia and/or thrombocytopenia. [b]Worst grade per patient. [c]One patient had a pre-existing grade 1 thrombocytopenia.

early disease progression (within 4 weeks of Carzelesin administration) in two patients and lost to follow-up in one patient. Table 3 gives an overview of the Carzelesin myelotoxicity. Both neutropenia and, to a lesser degree, thrombocytopenia were dose limiting, showing a high interpatient variability at all dose levels from 130 $\mu$g m$^{-2}$ onwards and causing dose-delay and/or reduction in several courses (Table 2). Consequently, the dose for further phase II studies was determined taking into account not only the level of myelotoxicity but also the actual dose intensity per course. At the first two dose levels, no grade 3 or 4 haematological toxicities were observed. At the dose levels 96 and 130 $\mu$g m$^{-2}$, two patients had grade 4 myelotoxicity. The intended dose intensity (IDI) could be administered up to the dose level 130 $\mu$g m$^{-2}$. At 150 $\mu$g m$^{-2}$, two patients out of six evaluable patients developed grade 4 myelotoxicity: the first patient, with renal cancer, pretreated with interferon, IL6 and vinblastine, developed during the first course grade 4 neutropenia and thrombocytopenia, with a recovery on day 42 leading in the subsequent three courses to a reduction of dose to 130 $\mu$g m$^{-2}$; the second patient, with colorectal cancer pretreated with 5FU and leucovorin, experienced grade 4 neutropenia during the second course of treatment without recovery by day 29. This showed that at 150 $\mu$g m$^{-2}$ most patients (four out of six) receiving $\geq$ 2 courses were able to receive the treatment at the scheduled time and dose, which was not possible for the 170, 210, 250 and 300 $\mu$g m$^{-2}$ dose levels (Table 2).

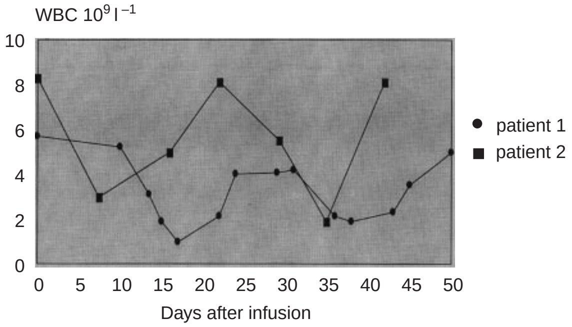

Moreover, at the dose level of 150 $\mu$g m$^{-2}$, the median received dose intensity was 37.5 $\mu$g m$^{-2}$ week$^{-1}$ (range: 27.3–37.5) and this was equivalent to the IDI at this dose level. For this reason 150 $\mu$g m$^{-2}$ every 4 weeks is recommended for further phase II studies.

The doses of Carzelesin were further escalated in order to define the MTD for a single course. This MTD was reached at 300 $\mu$g m$^{-2}$, at which two out of four patients experienced grade 4 neutropenia, of whom one patient also had grade 4 thrombocytopenia. This may be relevant for further studies in diseases where grade 4 leuco- and thrombocytopenia are not considered dose limiting (e.g. leukaemia).

**Table 4** Carzelesin: non-haematological toxicities in 31 evaluable patients[a]

| Toxicity | Worst CTC-grade per patient | | | |
|---|---|---|---|---|
| | 1 | 2 | 3 | 4 |
| Bilirubin | NA | 6 | 1 | 0 |
| Creatinine | 6 | 1 | 0 | 0 |
| Proteinuria | 3 | 0 | 0 | 0 |
| Nausea | 11 | 6 | 2 | 0 |
| Vomiting | 7 | 5 | 2 | 0 |
| Diarrhoea | 5 | 1 | 0 | 1[b] |
| Fatigue/asthenia | 3 | 9 | 3 | 0 |
| Fever | 4 | 5 | 2 | 0 |
| Pain at the infusion site | 9 | 2 | 0 | 0 |
| Mucositis | 7 | 1 | 0 | 0 |
| Anorexia | 5 | 5 | 0 | 0 |

NA = not applicable. [a]Toxicities seen for the dose levels: 150, 170, 210, 250, 300 μg m$^{-2}$ combined. [b]The relation to Carzelesin was unlikely.

Overall, the haematological toxicity was delayed in onset [median days to nadir were 12 (8–14) for neutrophils and 20 (15–26) for platelets], prolonged [median days to complete recovery were 28 (20–35) for neutrophils and 33 (24–56) for platelets] and appeared to be cumulative in some patients. Of note, from the dose level of 130 μg m$^{-2}$, a second drop in the leucocyte and neutrophil count after an apparent recovery was seen in 33% of evaluable patients and in 26% of evaluable courses (Figure 2). These ‘second nadirs’ caused a delay of treatment for the next courses and, consequently, a decrease in Carzelesin dose intensity.

Nine patients treated at different dose levels (from 130 μg m$^{-2}$) received red cell transfusions while on study. In general, these patients had mild pre-existing anaemia which worsened during treatment. No haemorrhagic episodes were noted during treatment with Carzelesin, in spite of the fact that thrombocytopenia was prolonged and dose limiting. One patient treated at the dose level of 250 μg m$^{-2}$ experienced an episode of febrile neutropenia. This patient was successfully treated with IV antibiotics and G-CSF.

### Non-haematological toxicities

Treatment with Carzelesin was generally well tolerated and non-haematological toxicities were infrequent and mild. Grade 1–2 fever, nausea/vomiting, mucositis, allergic reactions, anorexia and phlebitis were observed, none of which was clearly dose related. Table 4 summarizes non-haematological toxicities seen for the dose levels 150–300 μg m$^{-2}$ combined. A flush reaction was reported in five patients and, in two of these, drug administration was extended to 20 min.

### Pharmacokinetics–pharmacodynamics results

Of the 51 patients in this study, 31 were sampled for pharmacokinetic monitoring and evaluable for exploring pharmacokinetic–pharmacodynamic relationships. The results of the pharmacokinetic behaviour of Carzelesin and its major metabolites have been reported in detail elsewhere (Van Tellingen et al, 1998). In summary, the analytical method was sufficiently sensitive to establish a plasma concentration–time profile of Carzelesin from the first dose level of 24 μg m$^{-2}$ (Van Tellingen et al, 1994*a*). The peak plasma concentration at this dose was about 10 ng ml$^{-1}$ and the plasma level dropped to below 1 ng ml$^{-1}$ within 1 h after drug administration. At the highest dose level of 300 μg m$^{-2}$, the mean peak plasma levels of Carzelesin was 63 ng ml$^{-1}$, whereas the mean concentrations of the metabolites U-76,073 and U-76,074 were 18 and 3 ng ml$^{-1}$ respectively. From the dose level 96 μg m$^{-2}$, the concentration of the active metabolite U-76,074 ranged between 1 and 2 ng ml$^{-1}$ in the majority of the patients and, owing to these low plasma levels of U-76,074, it was not possible to calculate the elimination half-life of this metabolite, whereas the elimination half-life of Carzelesin was 23 ± 9 min (mean ± SD). Overall, there was a good correlation between the dose and the plasma AUC of Carzelesin, indicating linear pharmacokinetics.

The pharmacokinetic–pharmacodynamic relationships are depicted in Figure 3. The correlation between the AUC of Carzelesin vs the %decr in WBC, ANC or platelets was poor. However, the finding of detectable plasma levels of the active compound U-76,074 was usually associated with a greater than 60% decrease in ANC values. Only one patient was an exception to this finding: this patient, treated at 300 μg m$^{-2}$, experienced very modest toxicity, despite the fact that he also had the highest AUC of Carzelesin in this study. When this patient received the second course of treatment, the %decr in ANC was 82%.

### Antitumor activity

Eight patients were not evaluable for response. Thirty-four patients had progressive disease and nine patients had stable disease as best overall response.

### DISCUSSION

The CPI analogues are DNA minor groove binders containing a cyclopropyl group which mediates N$^3$-adenine covalent adduct formation in a sequence selective fashion (D’Incalci, 1994). Adozelesin (U-73,975), Carzelesin (U-80,244) and Bizelesin (U-77,779) are three agents of this family.

In this study, Carzelesin was given as a 4-weekly 10-min i.v. infusion to 51 patients with solid tumours. Haematological toxicity (neutropenia and to a lesser degree thrombocytopenia) was the only dose-limiting toxicity. It was delayed in onset (particularly for thrombocytopenia), prolonged and cumulative in some patients. The dose level with the best dose intensity for multiple courses was 150 μg m$^{-2}$ once every 4 weeks. This dose was selected for phase II

 © Cancer Research Campaign 1999

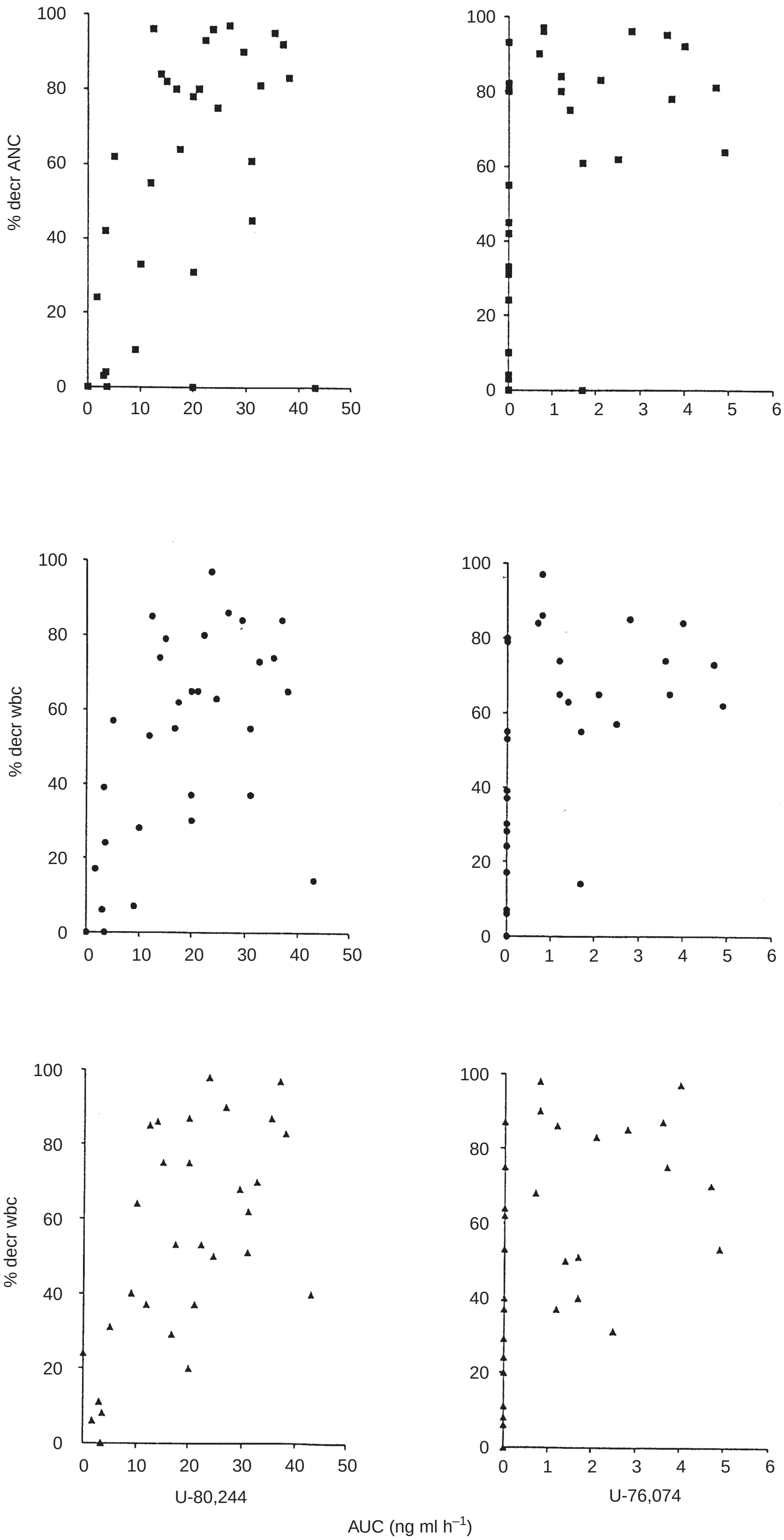

**Figure 3** Scatter plots of bone marrow toxicity depicted as %decr in white blood cells (WBC), granulocytes (ANC), and platelets vs. AUCs of Carzelesin and its active metabolite U-76,074

© Cancer Research Campaign 1999

studies which are presently ongoing in different tumour types within the framework of the EORTC-ECSG.

Carzelesin was also investigated in 25 patients with solid tumours using a daily times five schedule in another EORTC-ECSG study. With this schedule, the MTD was 40 μg $m^{-2}$ $day^{-1}$, and 35 μg $m^{-2}$ ('good risk' patients) and 30 μg $m^{-2}$ ('poor risk' patients), daily for 5 consecutive days every 4–5 weeks, were the recommended doses for phase II studies (Wolf et al, 1996). Concerning dose intensity on a mg $m^{-2}$ $week^{-1}$ basis, this schedule seems to offer no advantage over the day 1 every 4 weeks schedule studied in our study.

Carzelesin showed a pattern of myelotoxicity comparable with that of Adozelesin, the first CPI analogue which completed phase I and II evaluations (Shamdas et al, 1994; Fleming et al, 1994) but a less favourable pattern of myelotoxicity than another DNA-minor-groove alkylating agent, Tallimustine (distamycin derivative). In fact, Tallimustine given in a day 1 q 4 weeks schedule had a brief neutropenia as dose-limiting toxicity (Sessa et al, 1994).

The toxicity profile of Carzelesin is relatively specific to the bone marrow, providing a rationale for investigating this agent in haematological tumours, and in particular, in leukaemia patients.

Since the dose-limiting toxicity of Carzelesin is bone marrow suppression, we investigated the relationship between drug exposition (AUC of Carzelesin) and the percentage decrease in WBCs, ANC and platelets. Since CPI drugs exert their cytotoxicity by DNA adduct formation, we expected to find a relationship between drug exposure and toxicity. However, this relationship was very poor (Figure 3). In fact, the patient with the highest AUC observed in this series (viz. 43.2 ng $ml.h^{-1}$) experienced only very modest toxicity, whereas a marked toxicity was found in other patients with an AUC of less than 20 ng $ml.h^{-1}$. Since we already showed that there was a good correlation between the AUC of Carzelesin and the $C_{max}$ of Carzelesin and a similar good correlation between the AUC of Carzelesin and the AUC of the intermediate metabolite U-76,073 (Van Tellingen et al, 1998), we did not plot the %decr of the blood cells separately against these two parameters. On the other hand, since Carzelesin is a prodrug and U-76,074 the active CPI species, we explored the relationship between the AUC of this active compound and toxicity. Although there remains a substantial scatter in the data, it is noteworthy that a substantial decrease in ANC (i.e. > 60%) occurred mainly in patients whose plasma levels of U-76,074 exceeded 1 ng $ml^{-1}$ for some time. Only three patients with lower plasma levels of U-76,074 presented with a greater that 60% decrease in ANC values.

Although the use of U-76,074 levels to predict toxicity may seem attractive, it is important to realize that this parameter may be potentially flawed by two factors. Firstly, Carzelesin is converted to U-76,074 in the central blood compartment as well as intracellularly and, although this latter component may be even more important for the extent of myelotoxicity, it cannot be determined by measurement of plasma levels. Secondly, Carzelesin is readily converted to U-76,074 ex vivo if the sample is not immediately cooled in ice-water. Although much attention has been paid to warrant proper handling of the blood samples drawn from patients, it is not possible to exclude fully a contribution of ex vivo formation of U-76,074 in the levels determined by HPLC. It is obvious that pharmacokinetic–pharmacodynamic relationships need to be validated more extensively before they can be used in the clinic to predict toxicity.

The plasma level of the active metabolite U-76,074 extended above 1 ng $ml^{-1}$ for short periods of time in most patients receiving a dose of 130 μg $m^{-2}$ or higher. However, it is not clear which levels of U-76,074 are sufficiently high to induce an antitumour response in patients. Although the achieved concentrations are well above the concentration required to produce more than 50% tumour cell kill ($IC_{50}$ in in vitro cell cultures (Li et al, 1992), these levels are much lower than achieved in mice receiving this drug at the MTD (Van Tellingen et al, 1994*b*). Furthermore, recent results with tumour-bearing mice suggest that the dose–response curve is very steep (Houghton et al, 1995): indeed, when the dose was lowered to 50% of the MTD, the antitumour efficacy was significantly reduced. The profound haematologic toxicity of Carzelesin may obstruct the achievement of plasma levels required for antitumour activity. However, the final answer regarding the activity of this compound will be given by the ongoing phase II clinical studies.

### REFERENCES

D'Incalci M (1994) DNA-minor-groove alkylators, a new class of anticancer agents. *Ann Oncol* **5**: 877–878

EORTC-NDDO report (1992) Toxicology studies of Carzelesin (U-80244)

Fleming GF, Ratain MJ, O'Brien SM, Schilsky RL, Hoffman PC, Richards JM, Vogelzang NJ, Kasunic DA and Earhart RH (1994) Phase I study of adozelesin administered by 24-hour continuous intravenous infusion. *J Natl Cancer Inst* **86**: 368–372

Houghton PJ, Cheshire PJ, Hallman JDn and Houghton JA (1995) Therapeutic efficacy of the cyclopropylpyrroloindole, Carzelesin, against xenografts derived from adult and childhood solid tumours. *Cancer Chemother Pharmacol* **36**: 45–52

Hurley LH, Reynolds VL, Swenson DH, Petzold GL and Scahill TA (1984) Reaction of the antitumor antibiotic CC-1065 with DNA: structure of a DNA adduct with DNA sequence specificity. *Science* **226**: 843–844

Jonkman-de Vries JD, de Graaff-Teulen JA, Henrar REC, Kettenes-van-den-Bosch JJ, Bult A and Beijnen JH (1994) Pharmaceutical development of a parenteral formulation of the novel antitumour agent Carzelesin (U-80,244). *Invest New Drugs* **12**: 303–314

Lee CS and Gibson NW (1991) DNA damage and differential cytotoxicity produced in human carcinoma cells by CC-1065 analogues, U-73,975 and U-77,779. *Cancer Res* **51**: 6586–6591

Li LH, DeKoning TF, Kelly RC, Krueger WC, McGovren JP, Padbury GE, Petzold GL, Wallace TL, Ouding RJ, Prairie MD and Gebhard I (1992) Cytotoxicity and antitumour activity of Carzelesin, a prodrug cyclopropylpyrroloindole analogue. *Cancer Res* **52**: 4904–4913

McGovren JP, Clarke GL, Pratt EA and DeKoning TF (1984) Preliminary toxicity studies with the DNA-binding antibiotic, CC-1065. *J Antibiot (Tokyo)* **37**: 63–70

Reynolds VL, Molineux IJ, Kaplan DJ, Swenson DH and Hurley LH (1985) Reaction of the antitumor antibiotic CC-1065 with DNA location of the site of thermally induced strand breakage and analysis of DNA sequence specificity. *Biochemistry* **24**: 6228–6237

Sessa C, Pagani O, Zurlo MG, de Jong J, Hofmann C, Lassus M, Marrari P, Strolin Benedetti M and Cavalli F (1994) Phase I study of the novel distamycin derivative tallimustine (FCE 24517). *Ann Oncol* **5**: 901–907

Shamdas GJ, Alberts DS, Modiano M, Wiggins C, Power J, Kasunic DA, Elfring GL and Earhart RH (1994) Phase I study of adozelesin (U-73975) in patients with solid tumours. *Anticancer Drugs* **5**: 10–14

Van Tellingen O, Pels EM, Henrar REC, Schaaf LJ, Padbury GE, Beijnen JH and Nooijen WJ (1994*a*) Fully automated high-performance liquid chromatographic method for the determination of Carzelesin (U-80,244) and metabolites (U-76,073 and U-76,074) in human plasma. *J Chromatogr* **652**: 51–58

Van Tellingen O, Beijnen JH, Nooijen WJ, van Oene S, Henrar REC, Schaaf LJ and Padbury GE (1994*b*) Plasma pharmacokinetics of Carzelesin (U-80,244) and metabolites (U-76,073, U-76,074) in mice and rats. *Ann Oncol* **5**(suppl 5): 171 (Abstract)

Van Tellingen O, Punt CJA, Awada A, Wagener DJT, Piccart MJ, Groot Y, Schaaf LJ, Henrar REC, Nooijen WJ and Beijnen JH (1998) A clinical pharmacokinetics study of Carzelesin given by short-term intravenous infusion in a phase I study. *Cancer Chemother Pharmacol* **41**: 377–384

*© Cancer Research Campaign 1999*

Warpehoski MA and Hurley LH (1988) Sequence selectivity of DNA covalent modification. *Chem Res Toxicol* **1**: 315–333

Wolf I, Bench K, Beijnen JH, Bruntsch U, Cavalli F, de Jong J, Groot Y, Van Tellingen O, Wanders J and Sessa C (1996) Phase I clinical and pharmacokinetic study of Carzelesin (U-80,244) given on a daily × 5 schedule. *Clin Cancer Res* **2**: 1717–1723

© Cancer Research Campaign 1999
